# Supplementary figures and images for: The interplay of seizures-induced axonal sprouting and transcription-dependent Bdnf repositioning in the model of temporal lobe epilepsy
Source: PLoS One. 2021 Jun 4;16(6):e0239111. doi: 10.1371/journal.pone.0239111 (PMC8177504; doi:10.1371/journal.pone.0239111)

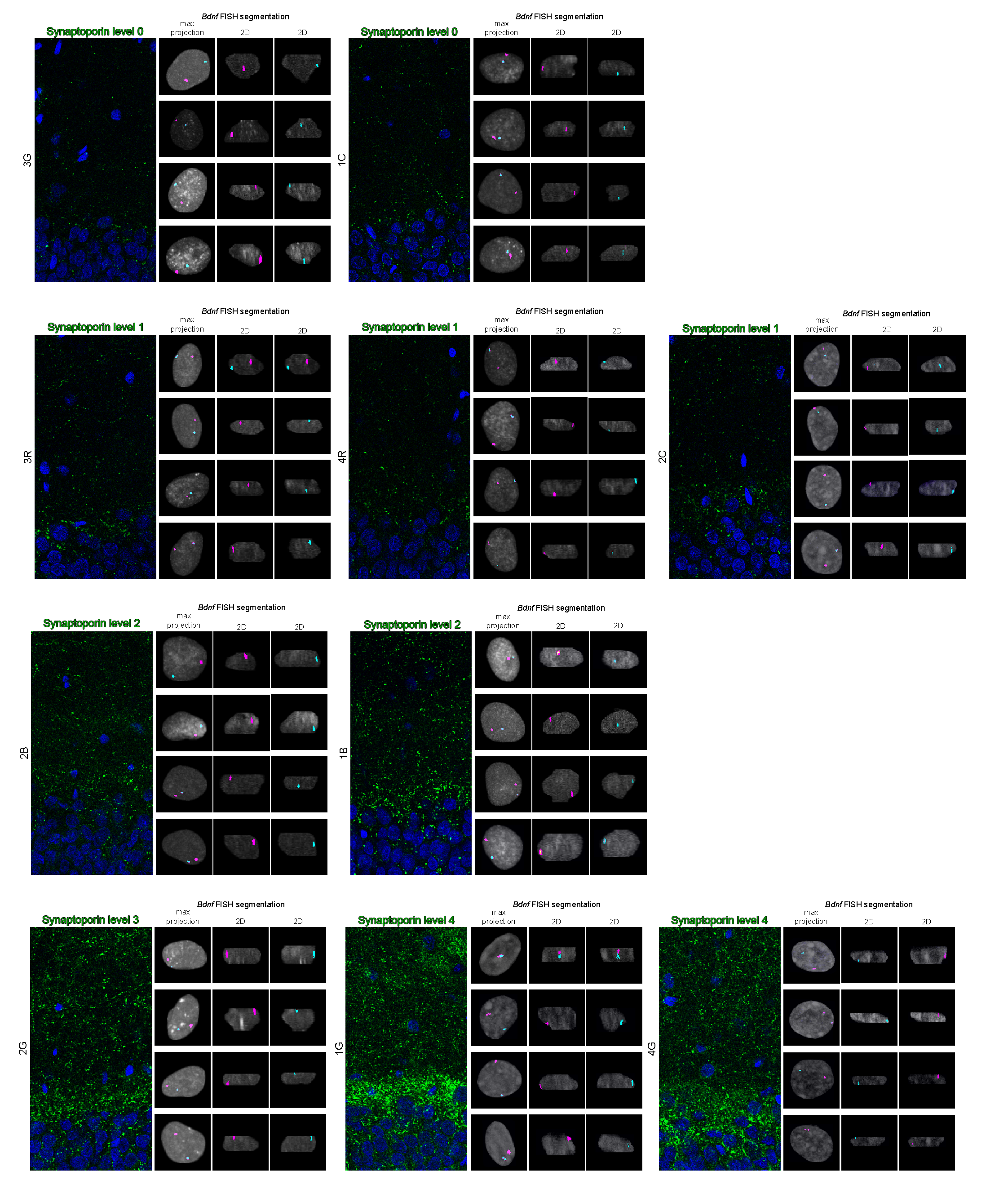

Supplement: S1 Fig — Mossy fiber sprouting was verified by immunofluorescent staining for synaptoporin (left panels, depicted in green) in the molecular layer of DG region of the hippocampus. Representative pictures from animals at 4 weeks after administration of kainate are shown. The right panels show images of nuclei of granular neurons acquired from the same animals. Hoechst 3342 staining for chromatin is shown in a grey scale and segmentation of FISH signals for Bdnf gene are shown in magenta and cyan. (TIF) [file pone.0239111.s001.tif]

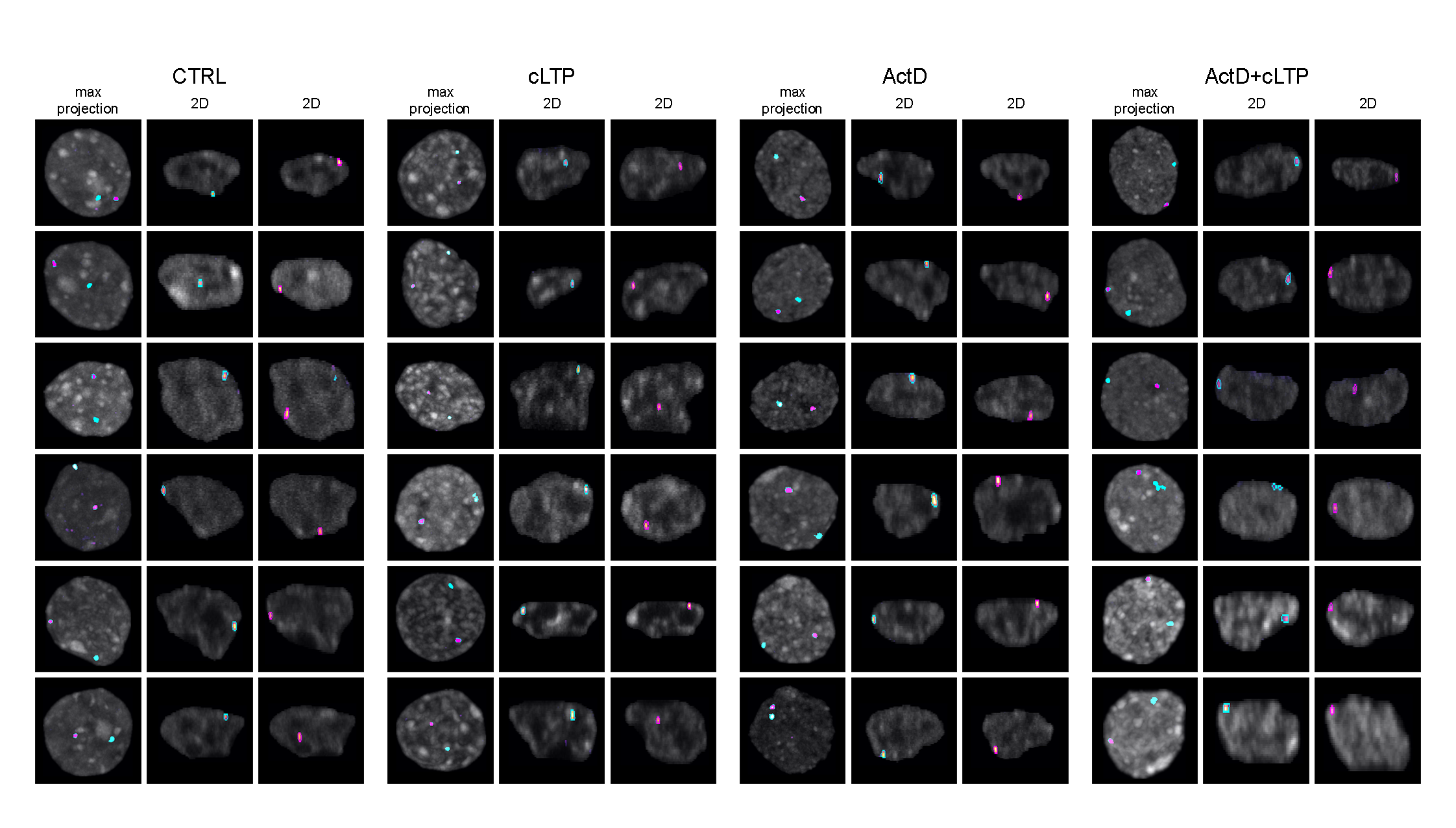

Supplement: S2 Fig — The representative pictures of the nuclei of hippocampal neurons incubated for 2 hours with DMSO vehicle (CTRL) or picrotoxin, forskolin, and rolipram (cLTP), incubated for 2 hours with Actinomycin D and 2 hours with DMSO (ActD) or picrotoxin, forskolin, and rolipram (ActD+cLTP). Hoechst 3342 staining for chromatin is shown in a grey scale and segmentation of FISH signals for Bdnf gene are shown in magenta and cyan. (TIF) [file pone.0239111.s002.tif]

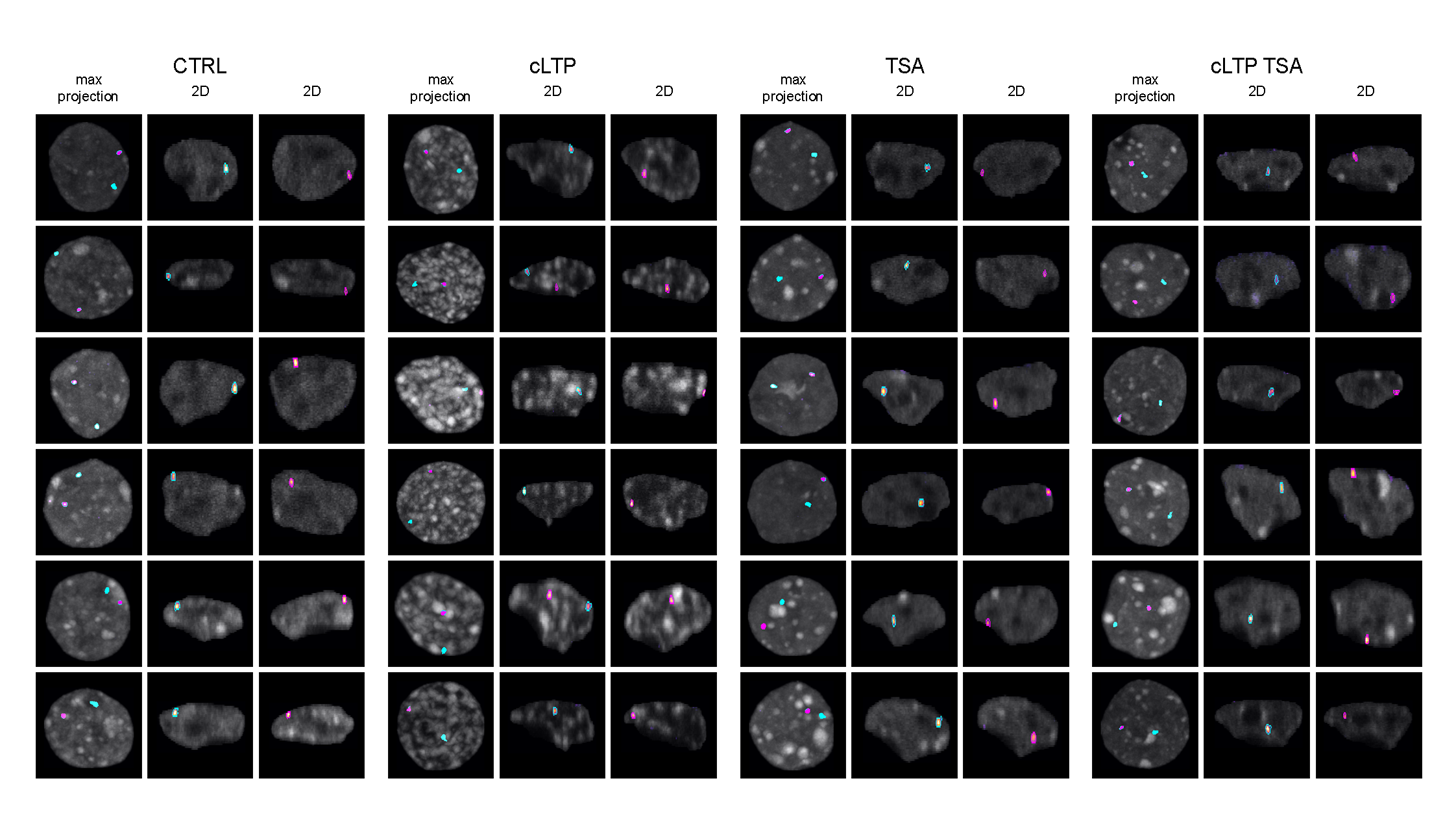

Supplement: S3 Fig — The representative pictures of the nuclei of hippocampal neurons incubated for 2 hours with DMSO vehicle (CTRL) or picrotoxin, forskolin, and rolipram (cLTP), incubated for 12 hours with Trichostatin A and 2 hours with DMSO (TSA) or picrotoxin, forskolin, and rolipram (cLTP TSA). Hoechst 3342 staining for chromatin is shown in a grey scale and segmentation of FISH signals for Bdnf gene are shown in magenta and cyan. (TIF) [file pone.0239111.s003.tif]

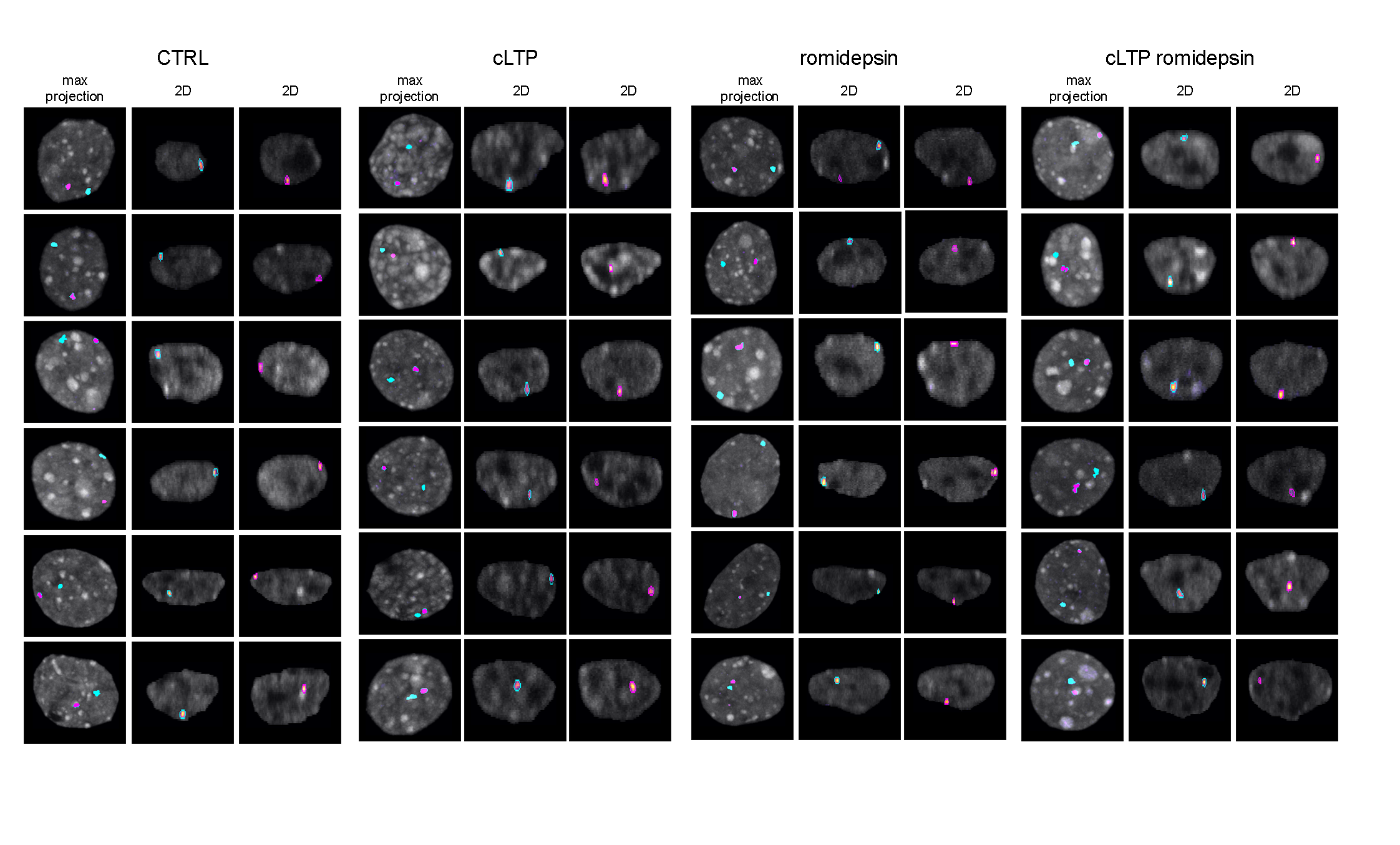

Supplement: S4 Fig — The representative pictures of the nuclei of hippocampal neurons incubated for 2 hours with DMSO vehicle (CTRL) or picrotoxin, forskolin, and rolipram (cLTP), incubated for 2 hours with romidepsin and 2 hours with DMSO (romidepsin) or picrotoxin, forskolin, and rolipram (cLTP romidepsin). Hoechst 3342 staining for chromatin is shown in a grey scale and segmentation of FISH signals for Bdnf gene are shown in magenta and cyan. (TIF) [file pone.0239111.s004.tif]
